# The Preparation and Properties of Volatile Tris(*N*‑Alkoxycarboxamidato)chromium(III) Complexes as Potential Single-Source Precursors for the MOCVD of Crystalline Chromium Oxide Thin Films

**DOI:** 10.1021/acsomega.5c00380

**Published:** 2025-05-20

**Authors:** Ji Hun Kim, Da Som Song, Sunyoung Shin, Ji Yeon Ryu, Duk-Young Jung, Jongsun Lim, Chang Gyoun Kim

**Affiliations:** † Thin Film Materials Research Center, 65680 Korea Research Institute of Chemical Technology, 141 Gajeong-ro, Yuseong-gu, Daejeon 34114, Republic of Korea; ‡ Department of Chemistry and Sungkyun Advanced Institute of Nanotechnology, Sungkyunkwan University, Suwon, Gyeonggi-do 16419, Republic of Korea

## Abstract

The synthesis, characterization, and vapor-phase growth of volatile Cr­(III) complexes as single-source precursors for Cr_2_O_3_ thin films are reported. A series of Cr complexes—Cr­(mdpa)_3_ (mdpa = *N*-methoxy-2,2-dimethylpropanamide) (**1**), Cr­(edpa)_3_ (edpa = *N*-ethoxy-2,2-dimethylpropanamide) (**2**), Cr­(empa)_3_ (empa = *N*-ethoxy-2-methylpropanamide) (**3**), and Cr­(mpa)_3_ (mpa = *N*-methoxypropanamide) (**4**)—were synthesized via salt elimination reactions between CrCl_3_·3THF and *N*-alkoxy carboxamidate salts. These complexes were characterized by Fourier transform infrared spectroscopy, elemental analysis, thermogravimetric analysis (TGA), single-crystal X-ray diffraction (SC-XRD), Hirshfeld surface analysis, powder X-ray diffraction (PXRD), and vapor pressure measurement. The crystal structure of complex **1** revealed a distorted octahedral geometry with a κ^2^ (O,O) binding mode. TGA demonstrated that complex **1** underwent weight loss at 132 °C and no residue remained at 500 °C. The enthalpy of vaporization of **1** was estimated to be 25.58 kJ/mol, making it an optimal precursor for Cr_2_O_3_ thin films. The XRD patterns of Cr_2_O_3_ films deposited on SiO_2_/Si substrates confirmed their crystalline nature, showing prominent peaks at 2θ = 33.5° and 41.5°. In addition, X-ray photoelectron spectroscopy validated the target Cr/O ratio, supporting the successful formation of Cr_2_O_3_ films.

## Graphical abstract

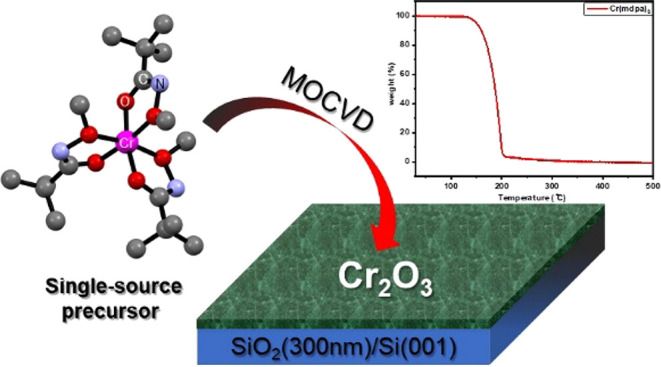

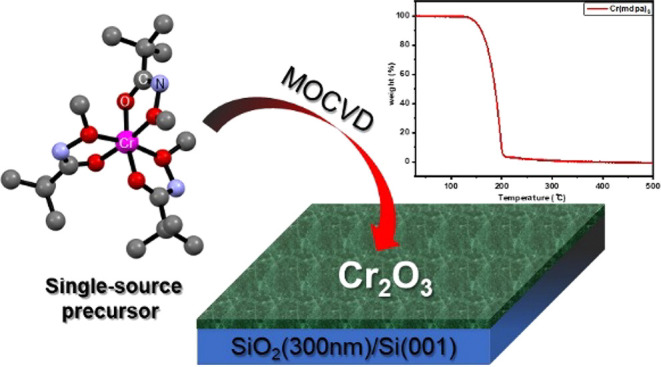

## Introduction

Chromium oxide (Cr_2_O_3_) has garnered significant attention owing to its tunable electrical,^1^ catalytic,^2^ and magnetic properties,^3,4^ making it a highly promising thin-film material for applications in gas sensors,^5–8^ photodetectors,^9^ protective coatings,^10^ catalysis,^11,12^ and electronic devices.^13,14^ In particular, Cr_2_O_3_ exhibits catalytic activity in oxidation reactions, including CO oxidation,^15^ due to the coexistence of Cr^3+^ and Cr^6+^, which facilitate redox cycling.^16^ Similar to MnO_2_ and CeO_2_, Cr_2_O_3_ utilizes multiple oxidation states to enhance catalytic activity. Additionally, Cr_2_O_3_ is known for its chemical stability and corrosion resistance, contributing to its durability in catalytic applications.^17^ Cr_2_O_3_ thin films are recognized as solar energy absorbers owing to their high solar absorption efficiency and low thermal transmittance.^18^ Furthermore, the conduction band edge of Cr_2_O_3_, which is comparable to that of perovskites, makes it suitable for use as a hole-blocking layer in optoelectronic devices.^19^ Because of their wide bandgap (approximately 3.1 eV), Cr_2_O_3_ thin films exhibit either *p*- or n-type semiconductor behavior depending on the growth conditions,^20^ making them particularly suitable for use in thin-film transistors and other semiconductor devices. Optimizing deposition techniques is crucial for exploiting the unique properties of Cr_2_O_3_ films in these applications.

Various chromium precursors, such as chromium hexacarbonyl (Cr­(CO)_6_),^21,22^ chromium acetylacetonate (Cr­(C_5_H_7_O_2_)_3_),^23,24^ and chromyl chloride (CrO_2_Cl_2_),^25^ have been used for the growth of Cr_2_O_3_ films using deposition techniques, such as plasma-enhanced chemical vapor deposition (PECVD),^21^ CVD,^23,25^ and atomic layer deposition (ALD).^24^ The selection of an oxygen source plays a critical role in facilitating oxide formation, ensuring stoichiometric accuracy, and producing high-quality films. Common oxygen sources include oxygen (O_2_) gas,^21,25^ O_2_ plasma,^26,27^ ozone (O_3_),^24,28^ which promote oxidation. For example, Mandol et al. investigated the ALD of Cr_2_O_3_ films using Cr­(acac)_3_ (acac = acetylacetonate) and O_3_ and demonstrated that the process facilitates uniform oxidation and ensures consistent film quality.^24^ Similarly, Kadari et al. demonstrated the effectiveness of O_2_ plasma in the efficient oxidation of Cr to form Cr_2_O_3_ films.^27^ The selection and appropriate use of oxygen sources are critical for producing high-quality Cr_2_O_3_ films because insufficient or inappropriate oxygen source can lead to structural defects and nonideal oxidation states.^29,30^ However, achieving precise control over the gas flow is challenging owing to issues related to maintaining stability and achieving a uniform distribution, which complicates the process. Single-source precursors offer significant advantages over conventional precursors by enabling early interactions between metal and oxygen, thereby improving uniformity of deposited films and eliminating the need for toxic or oxygen/moisture-sensitive gases.^31,32^ In addition, they reduce concerns about thermal behavior of the reactants and chemical incompatibility with other components or materials used in the process.^33^ For single-source precursors to be fully effective, they must possess low molecular weight, high volatility, and excellent thermal stability, which highlights the need for further research into optimizing precursor properties. This study reports the synthesis and characterization of four new Cr complexes as potential candidates for the deposition of Cr-based thin films via CVD and ALD: Cr­(mdpa)_3_ (**1**), Cr­(edpa)_3_ (**2**), Cr­(empa)_3_ (**3**), and Cr­(mpa)_3_ (**4**). Among these, complex **1**, with the highest volatility and thermal stability, was selected for the growth of Cr_2_O_3_ via metal–organic CVD (MOCVD), yielding films with excellent crystallinity. A key feature of this process is the formation of Cr_2_O_3_ films using a single-source precursor without additional reactants, highlighting the capabilities and high volatility of the precursor and making it a promising alternative to conventional precursors, such as Cr­(acac)_3_ and Cr­(tmhd)_3_ (tmhd = 2,2,6,6-tetramethyl-3,5-heptanedione).

## Results and Discussion

### Precursor Synthesis

Several chromium complexes were synthesized using *N*-alkoxy carboxamidate salts and CrCl_3_·3THF (Scheme 1). These complexes served as single-source precursors for Cr_2_O_3_ thin films. Carboxamidato chromium complexes minimized intermolecular interactions through modification of the pendant and amide groups of the carboxamide ligand, resulting in high volatility and low melting points.^34^

**Scheme 1:**
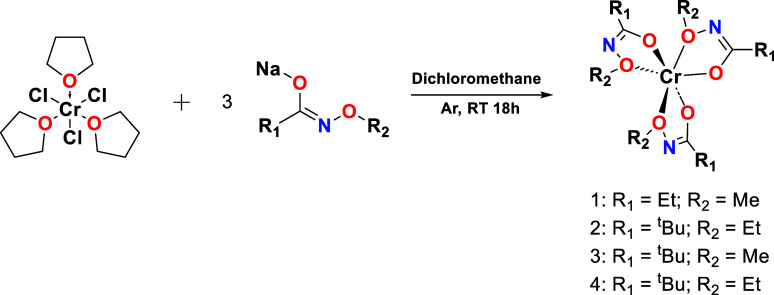
Scheme for the Synthesis of Cr­(III) Complexes **1–4**

Complexes **1–4** were synthesized via a salt elimination reaction between 1.0 equiv of CrCl_3_·3THF and 3.0 equiv of sodium-substituted N-alkoxy carboxamide ligands in dichloromethane at room temperature. The resulting complexes were blue (**1–3**) and blue-green (**4**) solids and were obtained in 71%, 78%, 49%, and 33% yields, respectively. As shown in Table 1, complexes **1**, **2**, and **4** were purified via sublimation at 50 °C under 0.5 Torr, 70 °C under 0.5 Torr, and 40 °C under 0.5 Torr, respectively. Complex **3** was purified via vacuum distillation at 90 °C under 0.8 Torr. The synthesized complexes had high solubility in organic solvents. Complexes **1** and **2** were stable under ambient conditions, whereas complexes **3** and **4** were stable under an inert atmosphere such as nitrogen or argon. The stability of the complexes was confirmed by recording their Fourier transform infrared (FT-IR) spectra after exposing them to ambient conditions for 1 day in open vials (Figures S1–S4).

**Table 1:** Physical and Thermal Properties of Cr Complexes, as Determined from Thermogravimetric Analysis (TGA)

Complex	Name	Formula	Molecular weight (g/mol)	Melting point (°C)	Sublimation temperature (°C)/pressure (torr)	Onset temperature for mass loss^a^ (°C)	Residual mass^b^ (%)
**1**	Cr(mdpa)_3_	C_18_H_36_CrN_3_O_6_	442.5	43	50/0.5	132	0
**2**	Cr(edpa)_3_	C_21_H_42_CrN_3_O_6_	484.6	47.5	70/0.5	121	3.7
**3**	Cr(empa)_3_	C_18_H_36_CrN_3_O_6_	442.5		90/0.8(distillation)	98	1.6
**4**	Cr(mpa)_3_	C_12_H_24_CrN_3_O_6_	358.3	33	40/0.5	112	12.7

a Temperature at 1% mass loss.

b Residual mass at 500 °C.

### Precursor Analysis

The synthesis and properties of the complexes were analyzed using FT-IR spectroscopy, elemental analysis (EA), single-crystal X-ray diffraction (SC-XRD), thermogravimetric analysis (TGA), and vapor pressure measurements.

#### FT-IR Spectroscopy

Because of the paramagnetic nature of complexes **1–4**, nuclear magnetic resonance (NMR) spectroscopy provided limited information. Therefore, the complexes were characterized by FT-IR spectroscopy. The FT-IR spectra of none of the complexes exhibited detectable N–H or O–H stretching frequencies in the vicinity of 3200 cm^–1^, as compared to the free ligand shown in Figures S5–S8. Additionally, the disappearance of the C=O band in the 1610 cm^–1^ region of the free ligand was observed, along with the appearance of a delocalized C=O band at the 1360 cm^–1^ region in the complexes. Consequently, the C–O stretching region exhibited two major bands at 1360 cm^–1^ and 1030 cm^–1^, corresponding to the delocalized C–O and alkoxy group C–O vibrations, respectively (Figure 1). The N–O stretching band of the free ligand appeared around 1230 cm^–1^, and it exhibited a red shift to the range of 1198–1172 cm^–1^ in the complexes. This shift can be attributed to the weakening of the N–O bond upon coordination of the oxygen atom to the chromium metal center, as well as the C–O bond. The peak for the C=N bond of the ligand was detected in the range 1549–1577 cm^–1^ in the FT-IR spectra of all complexes owing to the shifting of the resonance of the double bond after sodium substitution in the N-alkoxy carboxamide ligands. Moreover, the complexes **1–4** exhibited characteristic stretching peaks of alkyl groups in the range of 2900–2850 cm^–1^, with minor deviations depending on the specific alkyl group, such as t-butyl, ethyl, isopropyl, and methyl. Furthermore, the FT-IR spectra of all complexes exhibited a band corresponding to C–H bending at approximately 1450 cm^–1^, whereas weak peaks from 532 to 480 cm^–1^ corresponded to Cr–O asymmetric stretching, and those from 470 to 400 cm^–1^ correspond to Cr–O symmetric stretching, consistent with the IR peaks reported for Cr­(acac)_3_.^35^ These characteristic peaks were detected at identical wavenumbers in the FT-IR spectra of all four complexes, indicating consistent chromium–ligand bonding.

**Figure 1:**
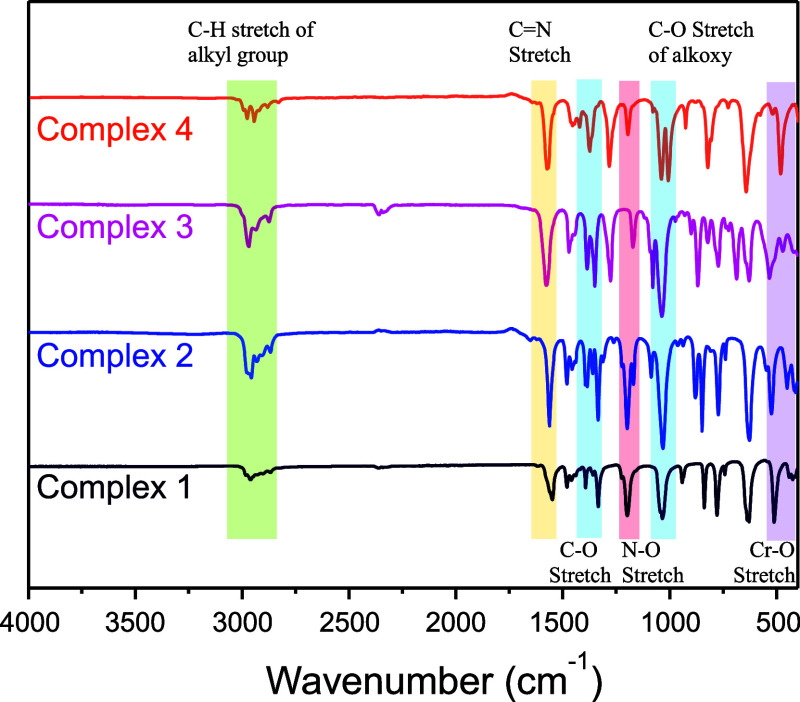
Infrared (IR) spectra of complexes **1–4**.

#### Crystal Structure

The solid-state structure of complex **1** was determined by X-ray crystallography. The blue crystals of complex **1** were obtained from a saturated hexane solution at −20 °C. The selected bond distances and angles for complex **1** are listed in Table 2, and detailed crystallographic data are provided in Table S1. Mononuclear complex **1** crystallized in the triclinic *P* 1 space group, with two molecules per unit cell (Figure S9). In the solid state, the complex exhibited a monomeric structure with a distorted octahedral geometry (Figure 2). The Cr atom was chelated by three monoanionic κ^2^ (O,O) binding modes, with the *N*-alkoxy carboxamidate ligands occupying all six coordination sites. The oxygen–metal center bond lengths (O–M) revealed two types of Cr–O bonds: Cr–O_short_ and Cr–O_long_. The Cr–O_short_ bonds had the following lengths: Cr(1)–O(1): 1.9378(16) Å, Cr(1)–O(3): 1.9192(15) Å, and Cr(1)–O(5): 1.9431(16) Å, whereas the Cr–O_long_ bonds had the following lengths: Cr(1)–O(2): 1.9854(17) Å, Cr(1)–O(4): 1.9844(16) Å, and Cr(1)–O(6): 1.9998(16) Å. This variation can be attributed to the different dative bonding interactions between the metal and oxygen atoms. Additionally, the Cr–O bond lengths are slightly broader range compared to those reported β-diketonate chromium complexes (1.942–1.959 Å).^36^ The delocalization induced by the substitution of the free ligand with the sodium form and its coordination to the chromium center resulted in two distinct groups of C–O bond lengths, ranging from 1.302 Å to 1.441 Å, as predicted by the IR analysis. The C=N bond of ligands had the following lengths: (1.292 Å–1.296 Å), which were consistent with typical C=N bond lengths reported in the literature. The angle between the axial O3 and O6 ligands was 172.33(7)°. The equatorial ligands O1, O2, O4, and O5 exhibited O–Cr–O bond angles ranging from 78.03(7)° to 95.22(7)°. The O–Cr–O bite angles were in the narrow range of 78.03(7)°–79.93(7)° and about 10° smaller than the reported Cr­(acac)_3_,^36^ giving the appearance of a five-membered ring. The dihedral angles (OCNO) within the five-membered chelate rings were 4.0(3)° for O(1)–O(2), 3.9(3)° for O(3)–O(4), and 0.8(3)° for O(5)–O(6). The intermolecular interaction revealed that the overall structure is stabilized by weak C–H···O and C–H···N hydrogen bonds with adjacent molecules (Figure S10). The H···O and H···N distances were determined to be 2.700 Å and 2.707 Å, respectively. Additional intermolecular interactions are discussed in the following section.

**Figure 2:**
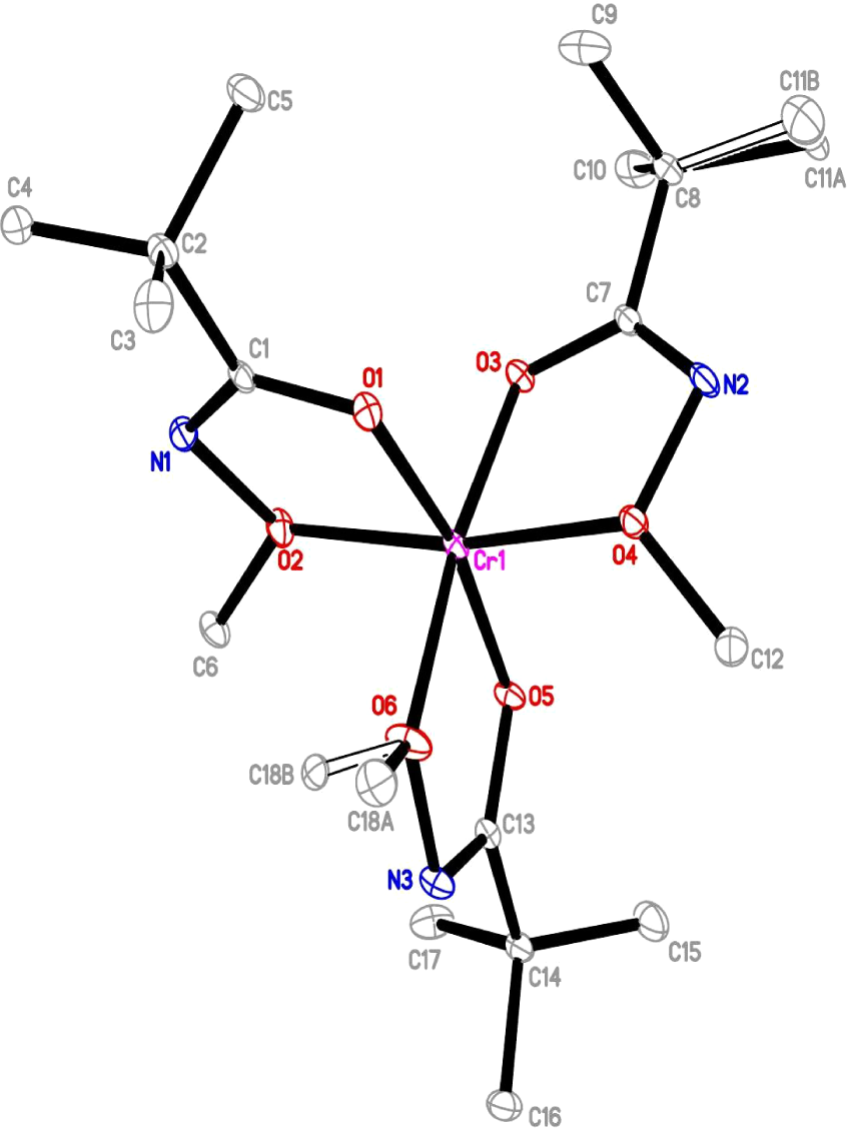
Crystal structure of complex **1**. Hydrogen atoms are omitted for clarity. Cr, pink; N, blue; O, red; and C, gray. Thermal ellipsoids of nonhydrogen atoms are shown at 25% probability.

**Table 2:** Selected Bond Distances (Å) and Angles (°) of Complex 1

Bond lengths (Å)			Bond angles (°)	
Cr(1)–O(1)		1.9192(15)	O(1)–Cr(1)–O(4)	91.95(7)
Cr(1)–O(3)		1.9431(16)	O(5)–Cr(1)–O(4)	95.22(7)
Cr(1)–O(5)		1.9431(16)	O(3)–Cr(1)–O(2)	94.31(7)
Cr(1)–O(2)		1.9854(17)	O(5)–Cr(1)–O(2)	94.56(7)
Cr(1)–O(4)		1.9844(16)	O(1)–Cr(1)–O(6)	92.70(7)
Cr(1)–O(6)	1.9998(16)		O(4)–Cr(1)–O(6)	93.91(7)
N(1)–C(1)		1.296(3)	O(2)–Cr(1)–O(6)	92.48(7)
N(2)–C(7)		1.292(3)	Ligand dihedral angles	
N(3)–C(13)	1.294(3)		O(2)–N(1)–C(1)–O(1)	–4.0(3)
O(1)–C(1)	1.311(3)		O(4)–N(2)–C(7)–O(3)	–3.9(3)
O(2)–C(6)		1.436(3)	O(6)–N(3)–C(13)–O(5)	0.8(3)
O(3)–C(7)		1.302(3)	Trans angles	
O(4)–C(12)	1.441(3)		O(1)–Cr(1)–O(5)	168.62(6)
O(5)–C(13)		1.312(3)	O(4)–Cr(1)–O(2)	169.24(7)
O(6)–C(18A)		1.388(5)	O(3)–Cr(1)–O(6)	172.33(7)
O(6)–C(18B)		1.438(7)	Bite angles	
			O(1)–Cr(1)–O(2)	79.10(7)
			O(3)–Cr(1)–O(4)	79.93(7)
			O(5)–Cr(1)–O(6)	78.03(7)

Consequently, complex **1** adopted a propeller-like form that minimized steric strains among the coordinated ligands, demonstrating pseudo-octahedral symmetry, which was similar to that reported for Cr­(acac)_3_^36^.

#### Hirshfeld Surface and Fingerprint Analysis

Hirshfeld surface (HS) analysis was conducted using CrystalExplorer 21.5 to examine the intermolecular interactions.^37^ Figure 3a represents the HS mapped over the normalized contact distance (*d*_norm_), with the range spanning from −0.5308 (red) to 1.4571 (blue) a.u. The red regions on the HS indicate close intermolecular contacts, especially prominent around hydrogen-bond donors/acceptors, while blue areas correspond to longer contact distances or noninteracting regions.

**Figure 3:**
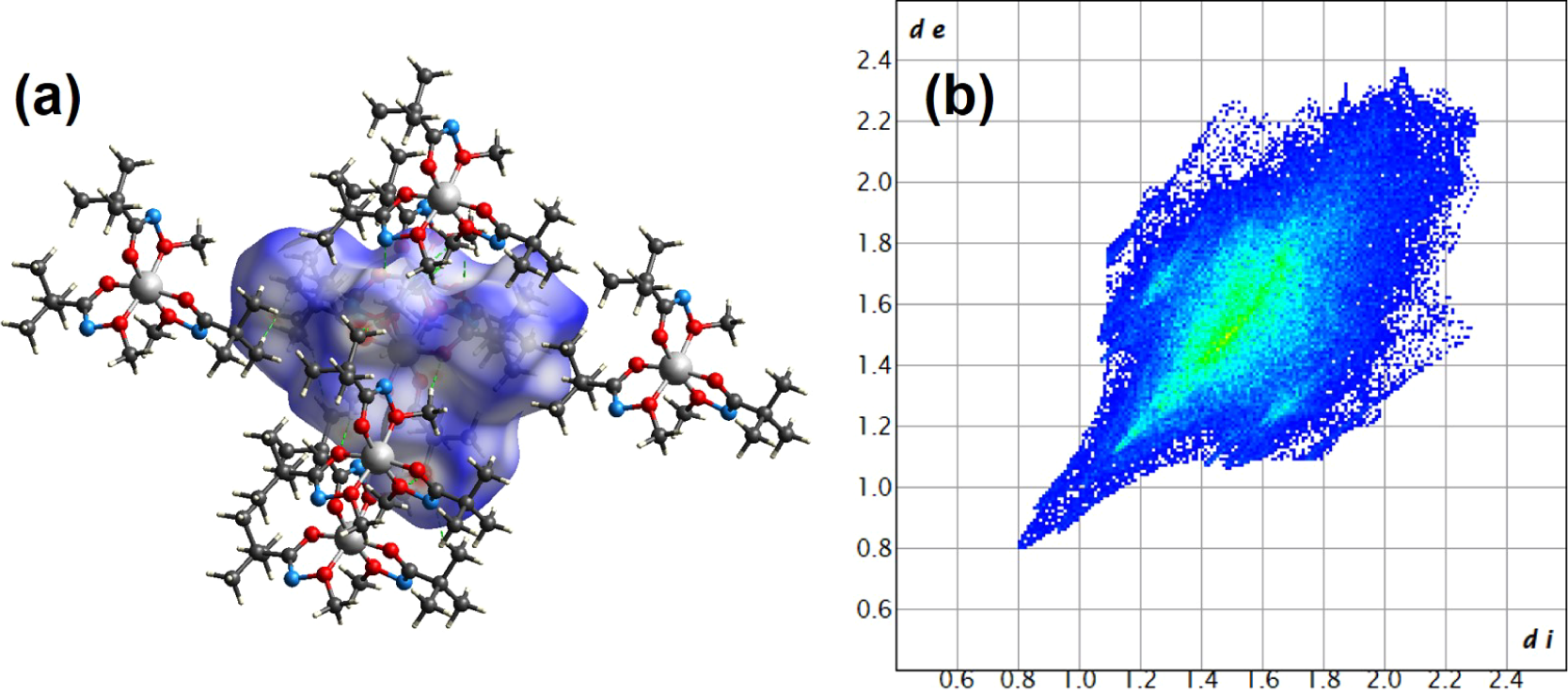
(a) Hirshfeld surface mapped over *d*_norm_ and (b) 2D fingerprint plots showing short intermolecular interactions for all atoms.

Two-dimensional fingerprint (FP) plots were generated to provide quantitative insight into the intermolecular interactions contributing to the overall crystal packing (Figure 3b). As shown in Figure S11, the dominant interaction is H···H, accounting for 84.3% of the total surface contacts, indicating that van der Waals interactions play a major role in the crystal packing. The C···H contacts contribute 0.6%, implying the presence of weak van der Waals or hydrogen-bonding interactions. O···H contacts contribute 8.0%, N···H interactions are also present at 7.0%, suggesting additional weak hydrogen bonding.

#### PXRD

The PXRD pattern of complex **1** was measured and compared with the calculated pattern from its SC-XRD structure in Mercury 4.2.0. As shown in Figure S12, the experimental PXRD pattern exhibits a strong correlation with the calculated pattern, with all major diffraction peaks similarly aligned in both 2θ positions and relative intensities. This correlation confirms that the bulk material is phase-pure and crystallographically consistent with the single-crystal structure.

Notably, at lower 2θ values, corresponding to larger *d*-spacings, the experimental peaks closely matched the calculated pattern, likely due to the rigid and bulky t-butyl groups in the ligand that maintained structural stability. However, after 10°, slight shifts toward lower angles were observed in the experimental pattern. This shift suggests the presence of residual tensile strain in the bulk crystallites, likely arising from differences in molecular packing or slight lattice expansion in the powder form.

#### Thermogravimetric Analysis

TGA measurements were conducted to investigate the volatility and thermal behavior of the complexes in the 30–500 °C range under a constant flow of nitrogen gas.

As shown in Figure 4 complexes **1**, **2**, and **4** exhibit identical thermal behavior, characterized by a single-step mass loss between 100 and 200 °C. The onset temperatures of mass losses were 132, 121, and 112 °C for complexes **1**, **2**, and **4**, respectively. Complex **1** exhibited the highest thermal stability and volatility, with no final residual mass at 500 °C. However, complexes **2** and **4** had final nonvolatile residual masses of 3.74% and 12.7%, respectively, at 500 °C. In contrast, complex **3** exhibited distinct thermal behavior, with a two-step mass loss process occurring at a lower onset temperature of 98 °C, resulting in a final nonvolatile residual mass of 1.6% at 500 °C. The slight weight loss observed after the major step at 220 °C in complexes **2–4** can be attributed to the partial decomposition of ligand fragments. These thermal decomposition profiles were consistent with trends reported for *N*-dialkoxy carboxamide groups, in which the homolytic cleavage of the nitrogen–oxygen bond occurs at temperatures above approximately 120 °C, generating alkoxy and alkoxyamidyl free radicals.^38^

**Figure 4:**
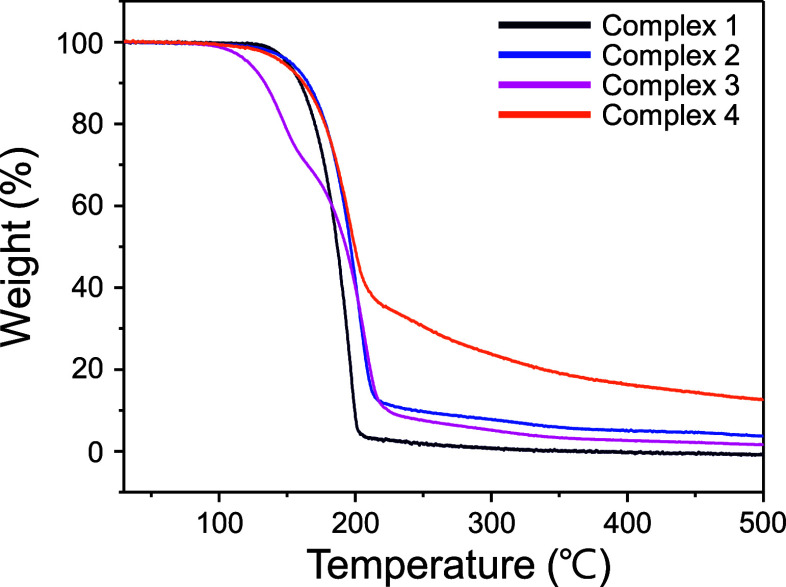
Thermogravimetric (TG) curves of complexes **1–4**. (heating rate = 10 °C/min under inert nitrogen atmosphere).

#### Vapor Pressure

Based on the previously measured TGA results, the precise volatility of **1** was determined through vapor pressure measurements. The vapor pressure measurement was conducted using the effusion method over a temperature range of 0–100 °C. To facilitate intuitive interpretation of the vapor pressure curve, Figure 5 presents the data with 1000 /*T* on the *x*-axis, while the ln *P* vs 1/*T* graph is provided in Figure S13. The vapor pressure–temperature correlation for **1** was determined using eq 1:

1 $$\ln P=\frac{-3076.8}{T}+5.4533$$

**Figure 5:**
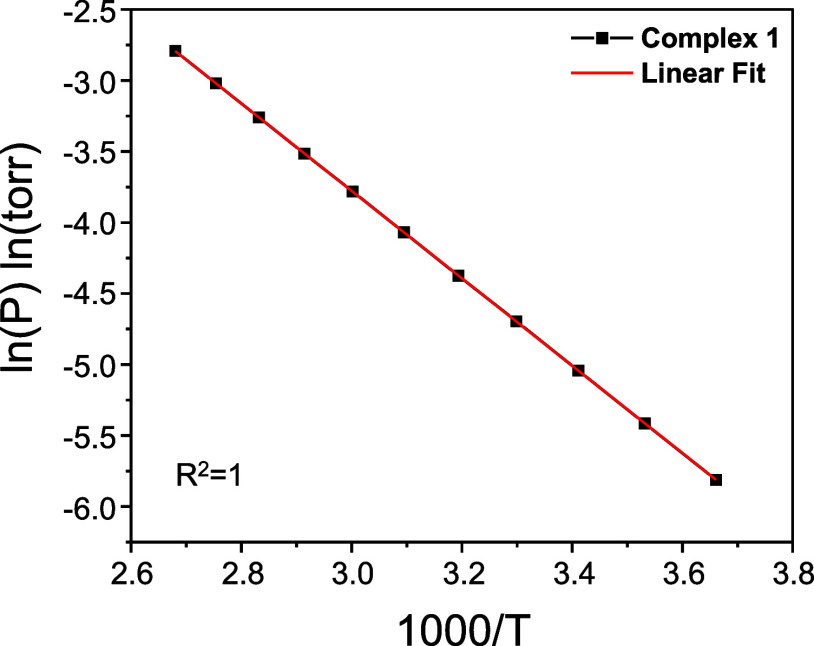
Vapor pressure measurement of complex **1**.

Here, *P* represents the pressure in torr, while *T* is the temperature in K. The sublimation enthalpy (Δ*H*_sub_) was calculated as 25.58 ± 0.01 kJ/mol based on the slope of eq 1, derived from the Clausius–Clapeyron equation. The temperature corresponding to 0.15 Torr was 145 °C, while the 1 Torr temperature was 291 °C. Comparing these values with previously reported Cr ALD/CVD precursors: CrCl_3_: Δ*H*_sub_ (245.20 ± 0.01 kJ/mol), 1 Torr temperature (680 °C); Cr­(acac)_3_: Δ*H*_sub_ (111.6 ± 3.0 kJ/mol), 1 Torr temperature (211 ± 10.0 °C); and Cr­(CO)_6_: Δ*H*_sub_ (63.3 ± 2.1 kJ/mol), 1 Torr temperature (35.95 ± 1.1 °C). Additionally, CrCl_3_ was measured using the TG-DSC-MS method,^39^ while Cr­(acac)_3_ and Cr­(CO)_6_ were analyzed using the TG transpiration method.^40^

### Growth and Characterization of Cr_2_O_3_ Films

The Cr­(mdpa)_3_ complex, a single-source precursor, was used to synthesize Cr_2_O_3_ films via MOCVD, as described in the Experimental Section. The structural, chemical, and morphological characteristics of the synthesized Cr_2_O_3_ films were characterized using SEM, XPS, XRD, and Raman spectroscopy. To optimize the growth conditions, Cr_2_O_3_ films were synthesized at 300 °C, 400 °C, 600 °C, 800 °C, and 900 °C, and their structural and chemical properties were investigated. The XPS survey spectra (Figure S14) revealed that at lower temperatures (<600 °C), significant residual carbon and nitrogen contamination was present, indicating incomplete decomposition of the precursor. Above 600 °C, the formation of Cr–O bonds became more evident, suggesting the formation of stable Cr_2_O_3_ structures. Raman spectroscopy (Figure S15) revealed that no characteristic peaks of Cr_2_O_3_ appeared at 600 °C, suggesting an amorphous structure, whereas weak peaks observed at 800 °C indicate the onset of crystallization. Based on these results, 900 °C was determined to be the optimal deposition temperature for achieving phase-pure and highly crystalline Cr_2_O_3_ films. Detailed structural and chemical analyses of the film grown under these conditions are presented in the following sections.

#### XPS

The chemical composition and oxidation states of Cr in the Cr_2_O_3_ films synthesized at 900 °C were analyzed by XPS. The survey spectrum confirms that the primary elements present on the film surface are Cr, O, and C (Figure S16a). To further investigate the distribution of carbon within the film, XPS depth profiling was performed on both the survey spectrum and the C 1s core-level spectrum. The results show that the C 1s peak intensity significantly decreases after sputtering, indicating that carbon is primarily present on the surface rather than being an intrinsic component of the film (Figure S16b). The Cr 2p core-level spectrum (Figure 6a) shows the presence of multiple oxidation states. The peaks at 575.9, 577.0, and 578.7 eV correspond to Cr­(OH)_3_, Cr_2_O_3_, and CrO_3_, respectively, indicating surface oxidation.^41–43^ The Cr 2p_1/2_ peak at 586.6 eV is separated from the Cr 2p_3/2_ peak by a binding energy difference of 9.6 eV, which is characteristic of the Cr^3+^ oxidation state in the Cr_2_O_3_.^44^ These results confirm that +3 is the dominant oxidation state of Cr in the film, with minor contributions from the +6 oxidation state of Cr and hydroxyl species owing to surface oxidation and hydroxylation. The O 1s XPS spectrum of the Cr_2_O_3_ thin film, as shown in Figure 6b, shows two fitted peaks:^42^ The peak at 530.5 eV corresponds to the lattice-bound oxygen in Cr_2_O_3_, and the peak at 531.6 eV corresponds to the oxygen of the hydroxyl group (−OH), indicating surface hydroxylation.^45^

**Figure 6:**
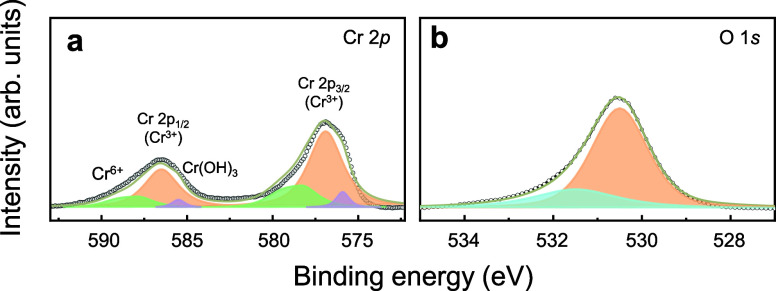
X-ray photoelectron spectroscopy (XPS) spectra of Cr_2_O_3_ thin films: (a) Cr 2p and (b) O 1s.

#### Raman Spectroscopy

Raman spectroscopy was used to evaluate the phase purity and crystallinity of the Cr_2_O_3_ thin films. The Raman spectrum in Figure 7a shows prominent peaks at 303, 350, 552, and 612 cm^–1^, corresponding to the characteristic A_1g_ and E_g_ vibrational modes of rhombohedral Cr_2_O_3_.^42,46^ Additionally, a peak at 520 cm^–1^, corresponding to SiO_2_/Si substrate, was observed. To eliminate substrate interference and confirm the presence of only Cr_2_O_3_ peaks, we used sapphire substrates in subsequent experiments; only Cr_2_O_3_ peaks were detected, as shown in Figure S17. These results confirm the presence of the rhombohedral phase of Cr_2_O_3_, thereby validating the phase purity of the film. The pronounced sharpness and intensity of these peaks indicate a well-ordered crystallographic structure, reflecting a high degree of crystallinity in the film.

**Figure 7:**
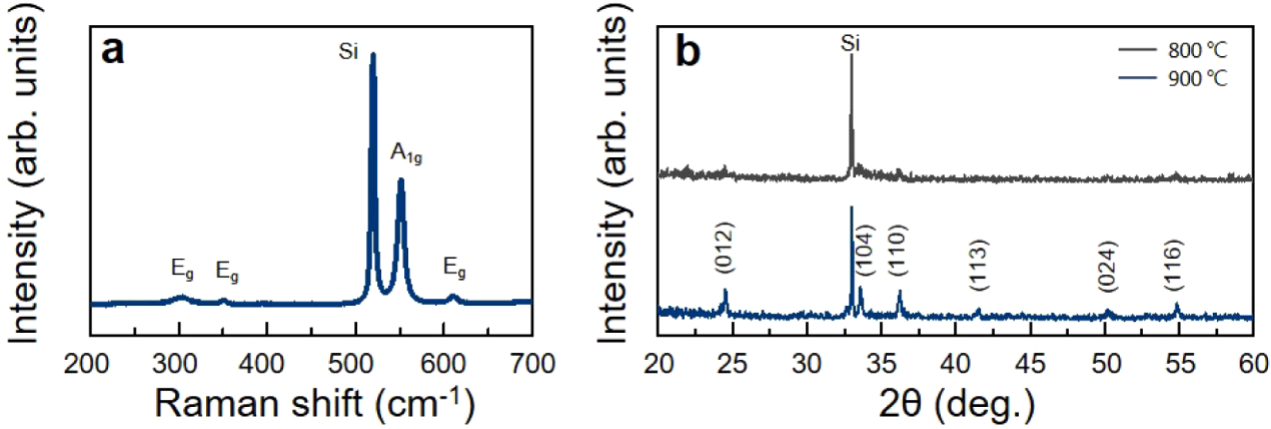
(a) Representative Raman spectrum and (b) XRD pattern of the Cr_2_O_3_ films synthesized at 800 and 900 °C.

#### XRD

In addition to Raman spectroscopy, XRD was used to elucidate the crystallographic structure of the Cr_2_O_3_ thin films. As shown in Figure 7b, the XRD patterns of films deposited at 800 and 900 °C reveal a clear difference in crystallinity. The film deposited at 900 °C displays well-defined diffraction peaks at 2θ = 24.5°, 33.5°, 36.2°, 41.5°, 50.1°, and 54.9°, corresponding to the (012), (104), (110), (113), (024), and (116) crystal planes of the rhombohedral Cr_2_O_3_ phase, respectively.^46,47^ The observed peak positions matched the reference standard (JCPDS 38-1479) for rhombohedral Cr_2_O_3_, confirming the correct identification of the Cr_2_O_3_ phase in the thin film. In contrast, the film obtained at 800 °C exhibits broadened and lower intensity peaks, indicating reduced crystallinity and incomplete phase formation. This comparison confirms that higher deposition temperature enhances the crystallinity and phase purity of the Cr_2_O_3_ films, with 900 °C identified as the optimal condition for high-quality film growth. Furthermore, the absence of secondary peaks indicates that the film was free of impurities or secondary phases, confirming the successful formation of a stoichiometric and phase-pure Cr_2_O_3_ film.

#### SEM

Figure 8 shows the top and cross-sectional SEM images of the Cr_2_O_3_ film. The analysis revealed that the film consisted of uniform, quasi-spherical particles. In the cross-sectional view (Figure 8a), the film exhibits a porous, irregular structure at the upper region, with smooth adhesion to the substrate at the bottom. The overall thickness of the film was approximately 1 μm. The columnar structure observed at the top indicates high-temperature growth, consistent with the Volmer–Weber growth mode. This growth mode is initiated when the surface energy of the substrate (γ_s_) is lower than the combined surface energies of the adatoms and interface (γ_a_ + γ_i_), promoting the formation of islands that eventually coalesce and grow vertically.^48,49^ In the top-view SEM image (Figure 8b), a uniform distribution of quasi-spherical particles with consistent shape and size is observed. The optical image (Figure S18) confirms that the film is uniformly distributed and fully covers the substrate.

**Figure 8:**
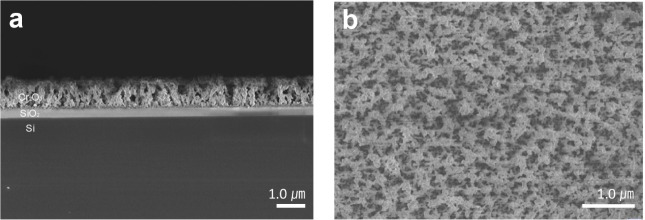
(a) Cross-sectional and (b) top-view scanning electron microscopy (SEM) images of Cr_2_O_3_ thin films on SiO_2_ (300 nm)/Si(001).

## Conclusion

In this study, we successfully synthesized and characterized four homoleptic single-source Cr complexes containing the *N*-alkoxy carboxamidate ligands: Cr­(mpda)_3_, Cr­(edpa)_3_, Cr­(empa)_3_, and Cr­(mpa)_3_. The volatility, thermal stability, and sublimation temperatures of these complexes indicate their strong potential as precursors for vapor-phase deposition processes. Among them, Cr­(mpda)_3_ exhibited the most promising properties for the production of high-quality Cr_2_O_3_ thin films. Using Cr­(mpda)_3_ as a single-source precursor in MOCVD resulted in the formation of crystalline Cr_2_O_3_ films, with +3 as the dominant oxidation state of Cr, as confirmed using XPS, Raman spectroscopy, and XRD analyses. These results confirm that Cr­(mpda)_3_ is an effective precursor for producing high-quality, crystalline Cr_2_O_3_ films, and the other three complexes can also be effectively used. Furthermore, these results underscore the potential of *N*-alkoxy carboxamide ligands to stabilize volatile chromium complexes, facilitating their broader application in thin-film deposition processes. This study provides a solid foundation for future investigations of group 6 element-based precursors for the development of advanced thin-film materials.

## Experimental Section

### General Considerations

All manipulations were performed in a nitrogen or argon atmosphere using standard Schlenk techniques or in a glovebox. Solvents were purified using a solvent purification system (Innovative Technology, PS-MD-4). All reagents were purchased from Sigma-Aldrich, Alfa Aesar, and TCI. A familly of *N*-alkoxy carboxamide ligands, edpaH, empaH, mpaH, and mdpaH, were synthesized following a reported procedure.^50^ Na­(mdpa) and Na­(edpa) were prepared using a modified method;^51^ Na­(empa) and Na­(mpa) were prepared using the same method described in the literature. CrCl_3_·3THF was synthesized by modifying the reported methods.^52^ IR spectra were recorded in the range of 4000–400 cm^–1^ using an FT-IR spectrophotometer (Shimadzu, IRSpirit). EA was performed using a CHNS analyzer (Thermo Scientific, Flash 2000). TGA was performed under an inert nitrogen atmosphere at a heating rate of 10 °C min^–1^ using a thermogravimetric analyzer (Netzsch, TG 209 F3TG). The structure of Cr­(mdpa)_3_ was confirmed using an SC-XRD (Bruker, SMART APEX II).

### Preparation of Starting Materials and Chromium Complexes 1–4

CrCl_3_ (9.5 g, 60 mmol) was dissolved in 100 mL of tetrahydrofuran in a round-bottom flask. A catalytic amount (0.01 g) of Zn dust was added to the reaction mixture and stirred overnight at room temperature. After completion of the reaction, 100 mL of dichloromethane was added to the flask, resulting in a deep purple solution. The solids were separated by filtration, and the filtrate was dried to obtain the desired product (CrCl_3_·3THF). Yield: 22.1 g (98.3%). IR (attenuated total reflectance (ATR), cm^–1^): 578, 681, 849, 919, 956, 1009, 1040, 1176, 1242, 1340, 1449, and 2978.

#### Synthesis of Cr­(mdpa)_3_ (**1**)

CrCl_3_·3THF (0.3747 g, 1 mmol) was dissolved in 40 mL of dichloromethane in a Schlenk flask, followed by the slow addition of Na-mdpa (0.4596 g, 3 mmol). The reaction mixture was then stirred overnight at room temperature. After filtering the mixture to remove the solids, the filtrate was concentrated under vacuum, and a blue solid of tri­(*N*-methoxy-2,2-dimethylpropanamide) chromium was obtained, which was sublimated to yield the final product. Yield: 0.3142 g (71%). IR (ATR, cm^–1^): 424, 440, 510, 628, 636, 741, 778, 837, 941, 1033, 1198, 1334, 1359, 1392, 1460, 1480, 1549, and 2959. Anal. for C_18_H_36_N_3_CrO_6_—calculated (found): C, 48.86 (48.85); H, 8.20 (8.35); and N, 9.50 (9.45).

#### Synthesis of Cr­(edpa)_3_ (**2**)

CrCl_3_·3THF (0.3747 g, 1 mmol) was dissolved in 40 mL of dichloromethane in a Schlenk flask, followed by the slow addition of Na-edpa (0.5016 g, 3 mmol). The reaction mixture was then stirred overnight at room temperature. After filtering the mixture to remove solids, the filtrate was concentrated under vacuum, and a blue solid of tri­(*N*-ethoxy-2,2-dimethylpropanamide) chromium was obtained, which was sublimated to yield the final product. Yield: 0.3779 g (78%). IR (ATR, cm^–1^: 450, 523, 626, 738, 772, 848, 879, 937, 962, 1031, 1086, 1169, 1198, 1261, 1313, 1335, 1360, 1385, 1393, 1455, 1480, 1562, 2868, 2932, and 2957. Anal. for C_21_H_42_N_3_CrO_6_—calculated (found): C, 52.05 (51.79); H, 8.74 (8.74); and N, 8.67(8.34).

#### Synthesis of Cr­(empa)_3_ (**3**)

CrCl_3_·3THF (1.77 g, 4.72 mmol) was dissolved in 40 mL of dichloromethane in a Schlenk flask, followed by the slow addition of Na-empa (2.17 g, 14.16 mmol). The reaction mixture was then stirred overnight at room temperature. After filtering the mixture to remove solids, the filtrate was concentrated under vacuum, and a blue viscous liquid of tri­(*N*-ethoxy-2,2-methylpropanamide) chromium was obtained, which was distilled to yield the final product. Yield: 1.02 g (49%). IR (ATR, cm^–1^): 470, 532, 628, 687, 724, 772, 821, 867, 899, 972, 1036, 1078, 1172, 1276, 1350, 1385, 1470, 1577, 2874, and 2969. Anal. for C_18_H_36_N_3_CrO_6_—calculated (found): C, 48.86 (50.41); H, 8.20 (8.72); and N, 9.50 (9.97).

#### Synthesis of Cr­(mpa)_3_ (**4**)

CrCl_3_·3THF (0.3747 g, 1 mmol) was dissolved in 40 mL of dichloromethane in a Schlenk flask, followed by the slow addition of Na-mpa (0.3753 g, 3 mmol). The reaction mixture was then stirred overnight at room temperature. After filtering the mixture to remove the solids, the filtrate was concentrated under vacuum, and a green-blue solid of tri­(*N*-methoxy-2,2-propanamide) chromium was obtained, which was sublimated to yield the final product. Yield: 0.1182 g (33%). IR (ATR, cm^–1^): 480, 518, 642, 726, 821, 925, 1006, 1039, 1077, 1195, 1282, 1373, 1421, 1453, 1573, 2943, and 2976. Anal. for C_12_H_24_N_3_CrO_6_—calculated (found): C, 40.22 (39.79); H, 6.75 (6.72); and N, 11.73 (11.50).

### Crystallography

The powder X-ray diffraction (PXRD) was performed using a diffractometer (Rigaku, D/MAX-2200/PC) at 40 kV and 40 mA with Cu Kα radiation in a 2θ range of 5°–50°. A single crystal of complex **1** was obtained via crystallization from hexane at −20 °C. A suitably sized and high-quality crystal was coated with Paratone oil and mounted on a glass capillary. Reflection data were collected using a diffractometer (Bruker, SMART Apex II-CCD area detector) with graphite-monochromated Mo Kα radiation (λ = 0.71073 Å). The hemisphere of the reflection data was acquired as ω-scan frames at 0.3° per frame with an exposure time of 10 s per frame. The unit cell parameters were determined and refined using the APEX2 software.^53^ The data were corrected for Lorentz and polarization effects, and empirical absorption correction was performed using the SADABS program.^54^ The structures of the complexes were determined by direct methods and refined by full-matrix least-squares using the SHELXTL program package^55^ and Olex2,^56^ with anisotropic thermal parameters applied to all nonhydrogen atoms. The crystallographic data for complex **1** have been deposited in the Cambridge Crystallographic Data Center (CCDC 2375514). These data are available free of charge from the CCDC at www.ccdc.cam.ac.uk/data_request/cif.

### Vapor Pressure

The vapor pressure of 1 was measured in accordance with OECD Test Guideline 104 at the Korea Polymer Testing and Research Institute (Koptri).

### Film Growth

Cr_2_O_3_ films were deposited onto hydrophilic SiO_2_ (300 nm)/Si(001) and quartz substrates via MOCVD at 300–900 °C for 30 min. During MOCVD, argon gas (99.9999% purity) was introduced at a flow rate of 200 sccm and a pressure of 0.4 Torr to maintain an inert atmosphere and serve as the carrier gas. The solid precursor was placed upstream of the tube furnace. The chemical bonding states of the films were characterized by XPS using a photoelectron spectrometer (Thermo Fisher Scientific, K-Alpha) equipped with a 180° double-focusing hemispherical analyzer and a 128-element multichannel detection system. XPS spectra were collected with normal emission geometry using monochromatic Al Kα radiation (hν = 1486.6 eV), with a spot size of 400 × 400 μm^2^. A pass energy of 50 eV and a step size of 0.1 eV were used for data acquisition. The crystallinity and structure of the films were analyzed by Raman spectroscopy (NR 500, Sol Instruments). Additionally, XRD patterns were recorded using a diffractometer (Rigaku, D/MAX-2200/PC) operating at 40 kV and 40 mA with Cu Kα radiation in a 2θ range of 10°–80°. Surface morphology was examined by SEM (JSM-6700F, Sigma HDJEOL).

## Supplementary Material





## Notes

Crystallographic data for complex **1** (CIF)
X-ray crystallographic data and parameters for complex **1**; FT-IR spectra showing stability of complexes **1–4** under ambient conditions; FT-IR spectra for comparison of complex **1–4** with free ligand; packing, view, and 2D fingerprint of intermolecular interactions of complex **1**; X-ray diffraction pattern of **1**; vapor pressure measurement of ln *P* vs 1/T; XPS spectra (survey, Cr 2p and O 1s), Raman and XRD of Cr_2_O_3_ films synthesized at various temperatures; XPS depth profiling analysis (survey and C 1s) of the Cr_2_O_3_ film; Raman spectra of Cr_2_O_3_ film on sapphire substrate; optical image of Cr_2_O_3_ film on SiO_2_/Si (PDF)
