# Supplementary material for: The Preparation and Properties of Volatile Tris(N‑Alkoxycarboxamidato)chromium(III) Complexes as Potential Single-Source Precursors for the MOCVD of Crystalline Chromium Oxide Thin Films
Source: ACS Omega. 2025 May 20;10(21):21519–28. doi: 10.1021/acsomega.5c00380 (PMC12138604; doi:10.1021/acsomega.5c00380)
Supplement: Supplementary file 2 [file ao5c00380_si_002.pdf]

Supporting Information

The Preparation and Properties of Volatile Tris(*N*-alkoxycarboxamidato)chromium(III) Complexes as Potential Single-Source Precursors for the MOCVD of Crystalline Chromium Oxide Thin Films

Ji Hun Kim[a,b]‡, Da Som Song[a]‡, Sunyoung Shin[a], Ji Yeon Ryu[a], Duk-Young Jung[b], Jongsun Lim[a]*, Chang Gyoun Kim[a]*

[a]Thin Film Materials Research Center, Korea Research Institute of Chemical Technology, 141 Gajeong-ro, Yuseong-gu, Daejeon 34114, Republic of Korea

[b]Department of Chemistry and Sungkyun Advanced Institute of Nanotechnology, Sungkyunkwan University, Suwon 16419, Gyeonggi-do, Republic of Korea

Contents

Precursor analysis 4

Table S1. X-ray Crystallographic Data and Parameters **1**............................. 4

Figure S1. IR analysis of complex **1** for stability in ambient conditions 5

Figure S2. IR analysis of complex **2** for stability in ambient conditions 5

Figure S3. IR analysis of complex **3** for stability in ambient conditions 6

Figure S4. IR analysis of complex **4** for stability in ambient conditions 6

Figure S5. IR analysis for comparison of complex **1** ($Cr(mdpa)_3$), Na-mdpa and mdpaH...7

Figure S6. IR analysis for comparison of complex **2** ($Cr(edpa)_3$), Na-edpa and edpaH...7

Figure S7. IR analysis for comparison of complex **3** ($Cr(empa)_3$), Na-empa and empaH...8

Figure S8. IR analysis for comparison of complex **4** ($Cr(mpa)_3$), Na-mpa and mpaH...8

Figure S9. Packing of complex **1**. View down the (**a**) crystallographic *a*-axis, and (**b**) crystallographic *b*-axis...9

Figure S10. View of complex **1** along the b-axis. Dotted lines indicate the intermolecular interactions. (Cr, pink; N, blue; O, red; C, gray; and H, white) 9

Figure S11. 2D fingerprint plots in crystal packing of **1** (a) N...H (b) O..H (c) H...H (d) C...H...10

Figure S12. Experimental and calculated powder X-ray diffraction pattern of **1**...11

Figure S13. Vapor pressure measurement of lnP vs. 1/T................................11

Film analysis 12

Figure S14. XPS survey and core-level spectra (Cr 2p and O 1s) of Cr_2O_3 films synthesized at various temperatures: (**a**) single-source precursor, (**b**) 300 °C, (**c**) 400 °C, (**d**) 600 °C, (**e**) 800 °C, and (**f**) 900 °C...12

Figure S15. Raman spectra of Cr_2O_3 films synthesized at 600 °C and 800 °C...12

Figure S16. XPS depth profiling analysis of the Cr_2O_3 film synthesized at 900 °C: (**a**) survey spectrum, (**b**) C 1s depth profile……………………………...……..……13

Figure S17. Raman spectra of Cr_2O_3 film on sapphire substrate……………………13

Figure S18. Optical image of Cr_2O_3 film on SiO_2/Si……………….……………14

Table S1. X-ray Crystallographic Data and Parameters 1

$Cr(mdpa)_3$ (1)	
Empirical formula	C18 H36 Cr N3 O6
Formula weight	442.5
Temperature	100(1) K
Wavelength	0.71073 Å
Crystal system	Triclinic
Space group	P-1
Unit cell dimensions	a = 10.1866(5) Å
	b = 10.9684(5) Å
	c = 12.0493(6) Å
Volume	1157.51(10) $Å^3$
Z	2
Density (calculated)	1.270 Mg/m^3
Absorption coefficient	0.529 mm^{-1}
F(000)	474
Theta range for data collection	1.877 to 27.997°
Index ranges	-13<=h<=10, -14<=k<=14, -15<=l<=15
Reflections collected	20160
Independent reflections	5556 [R(int) = 0.0445]
Completeness to theta = 25.242°	99.70%
Absorption correction	Semi-empirical from equivalents
Max. and min. transmission	0.94 and 0.80
Refinement method	Full-matrix least-squares on F^2
Data / restraints / parameters	5556 / 24 / 287
Goodness-of-fit on F^2	1.12
Final R indices [I>2sigma(I)]	R1 = 0.0551, wR2 = 0.1265
R indices (all data)	R1 = 0.0642, wR2 = 0.1312
Largest diff. peak and hole	0.730 and -0.328 e.$Å^{-3}$

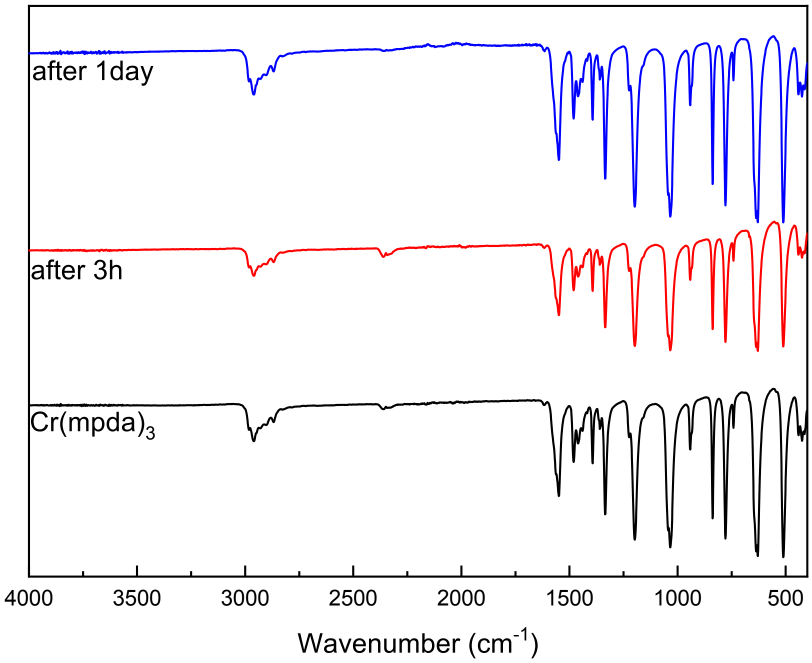

Figure S1. IR analysis of complex **1** for stability in ambient conditions

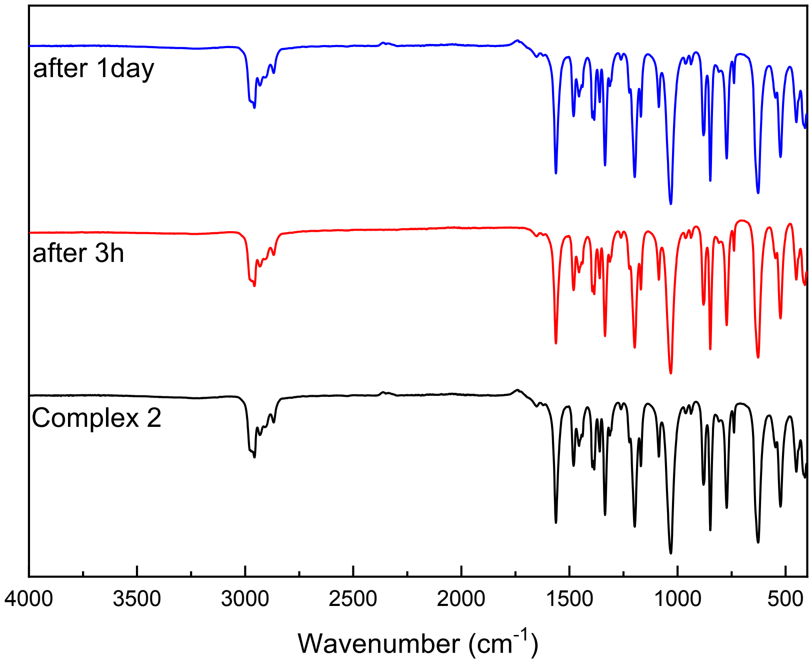

Figure S2. IR analysis of complex **2** for stability in ambient conditions

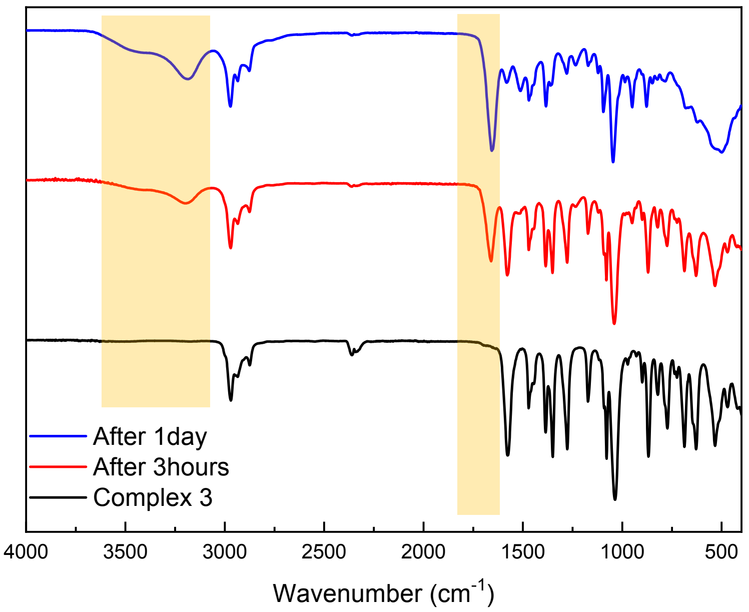

Figure S3. IR analysis of complex **3** for stability in ambient conditions

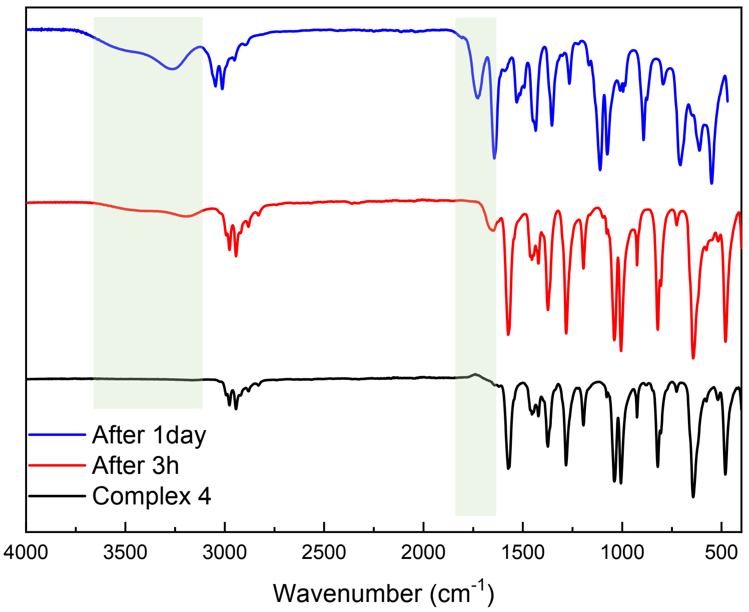

Figure S4. IR analysis of complex **4** for stability in ambient conditions

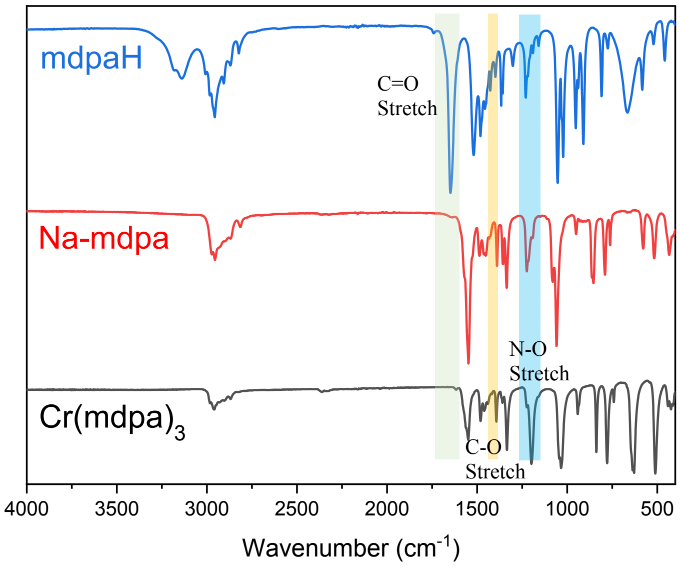

Figure S5. IR analysis for comparison of complex **1** (Cr(mdpa)$_3$), Na-mdpa and mdpaH

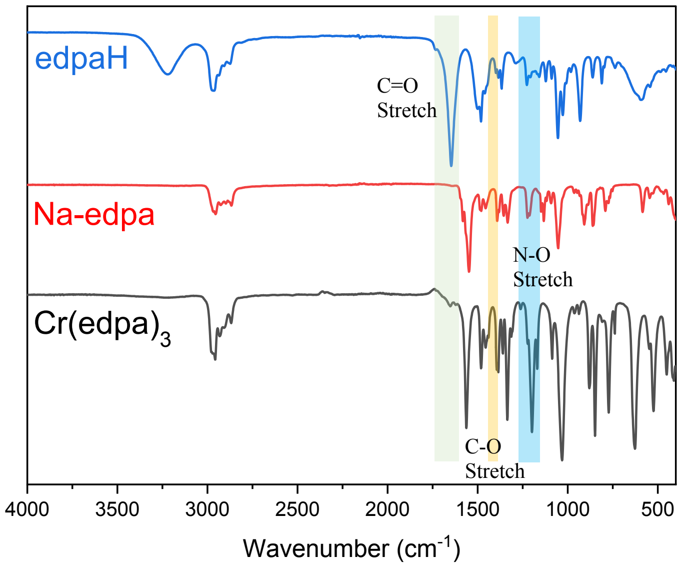

Figure S6. IR analysis for comparison of complex **2** (Cr(edpa)$_3$), Na-edpa and edpaH

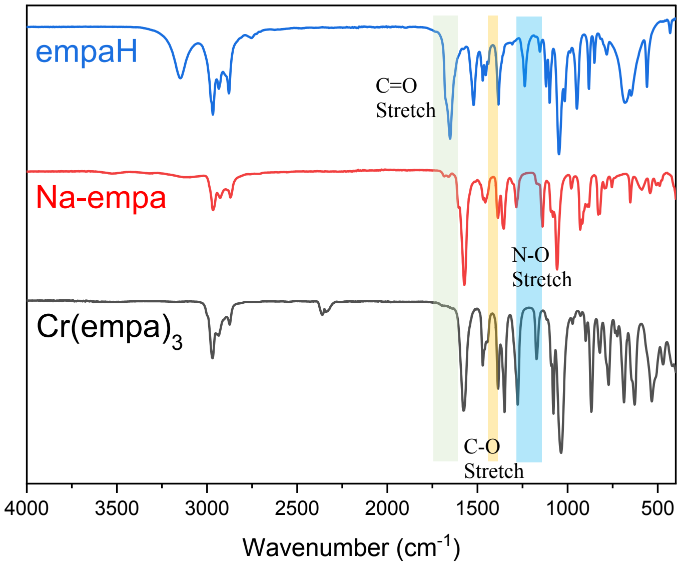

Figure S7. IR analysis for comparison of complex **3** (Cr(empa)$_3$), Na-empa and empaH

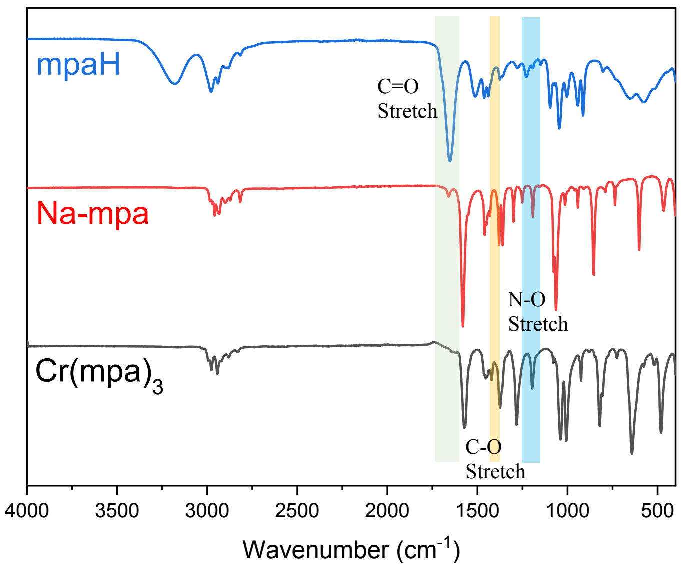

Figure S8. IR analysis for comparison of complex **4** (Cr(mpa)$_3$), Na-mpa and mpaH

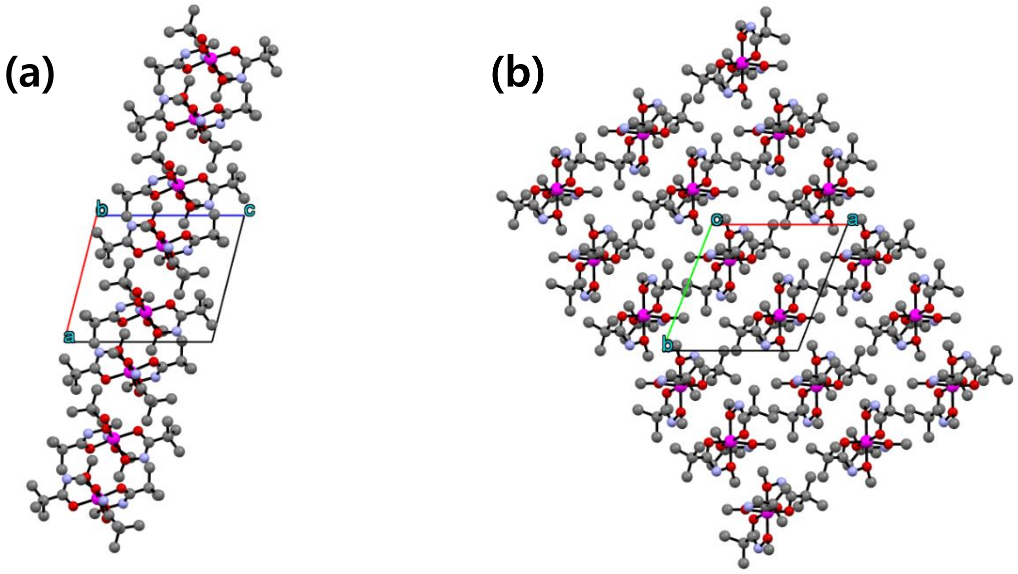

Figure S9. Packing of complex **1**. View down the (**a**) crystallographic *a*-axis, and (**b**) crystallographic *b*-axis.

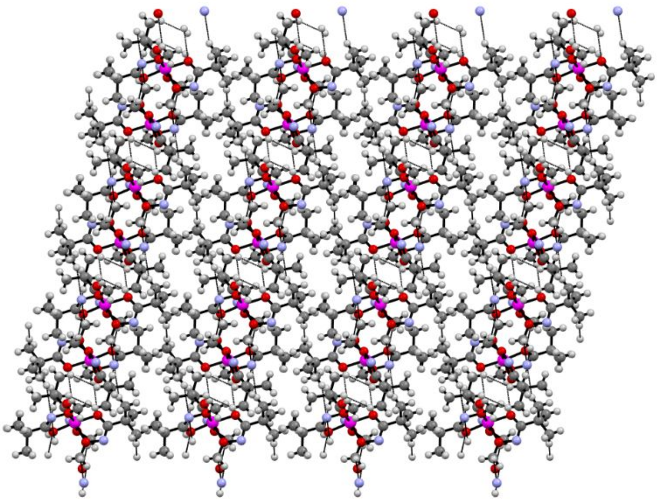

Figure S10. View of complex **1** along the b-axis. Dotted lines indicate the intermolecular interactions. (Cr, pink; N, blue; O, red; C, gray; and H, white)

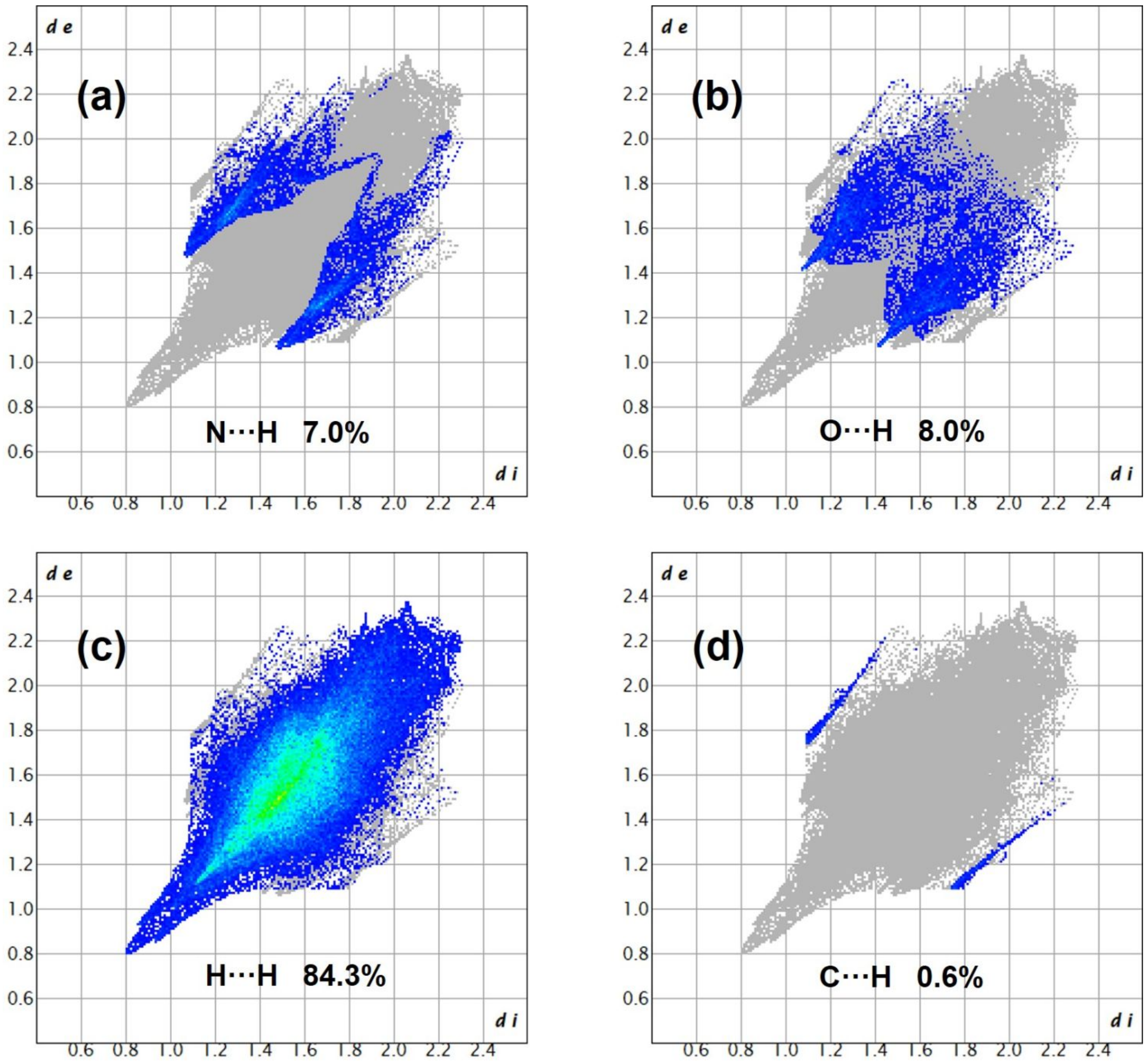

Figure S11. 2D fingerprint plots in crystal packing of **1** (a) N...H (b) O..H (c) H...H (d) C...H

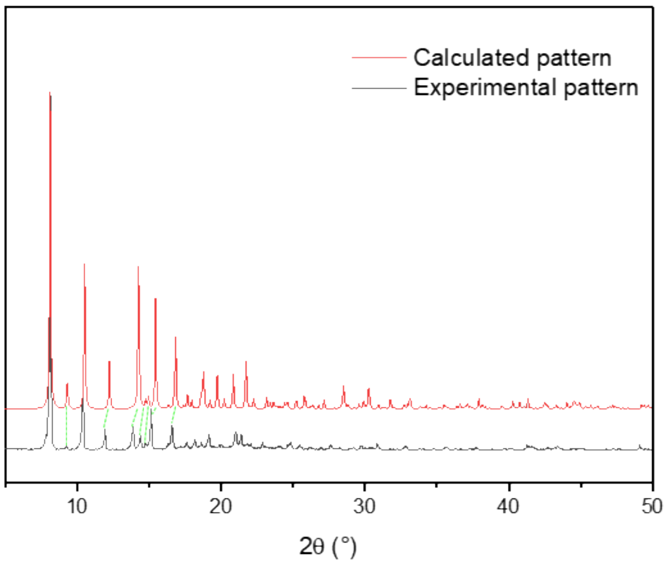

Figure S12. Experimental and calculated powder X-ray diffraction pattern of **1**

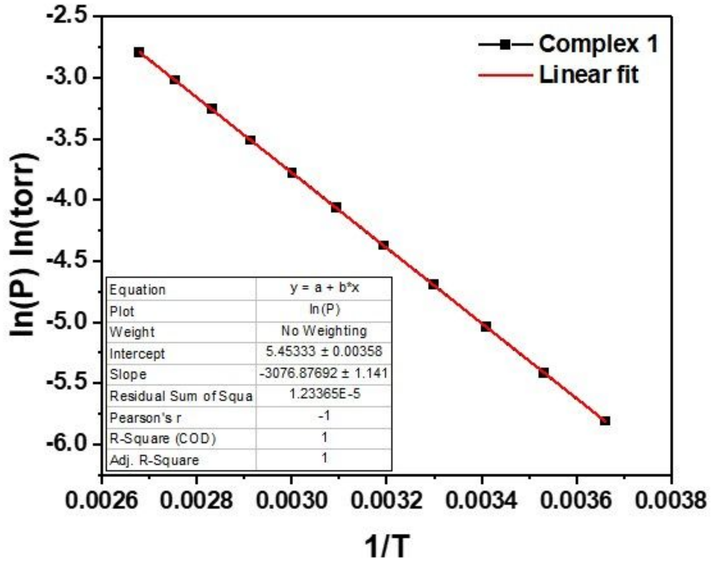

Figure S13. Vapor pressure measurement of lnP vs. 1/T

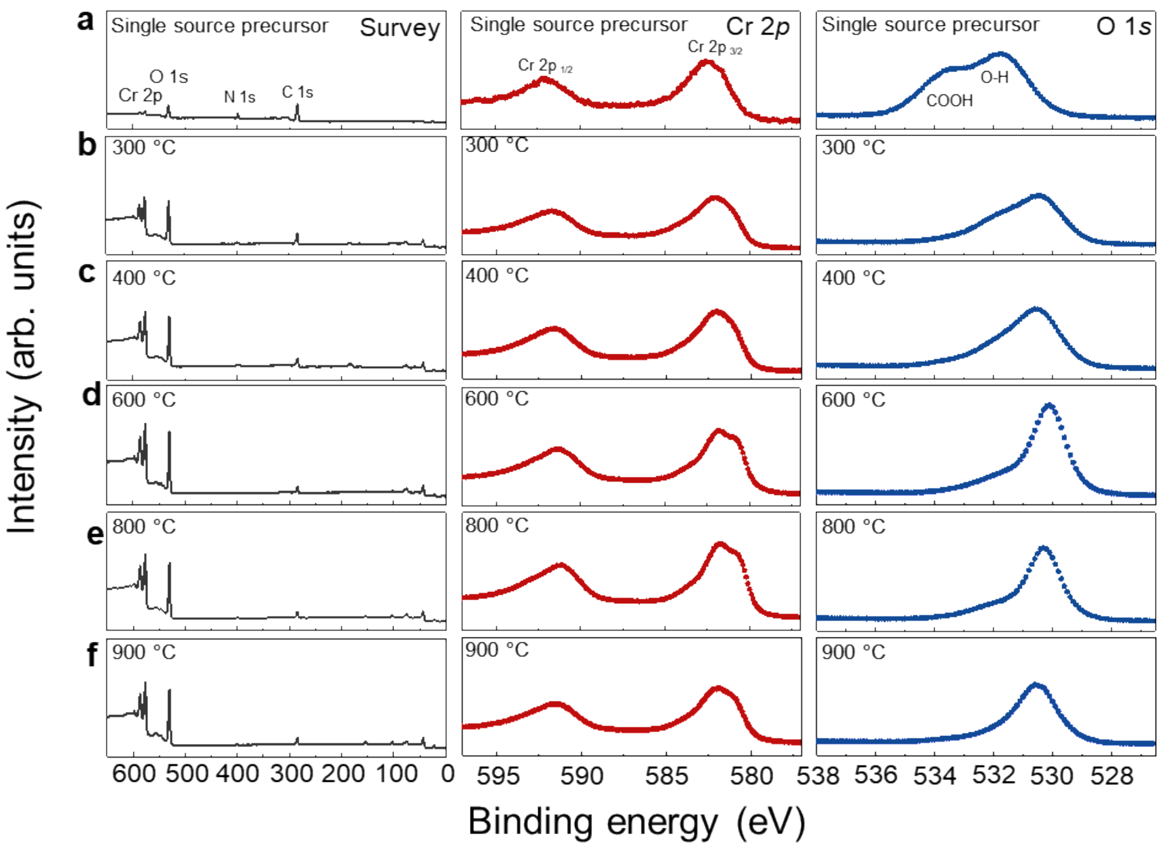

Figure S14. XPS survey and core-level spectra (Cr 2p and O 1s) of Cr_2O_3 films synthesized at various temperatures: (**a**) single-source precursor, (**b**) 300 °C, (**c**) 400 °C, (**d**) 600 °C, (**e**) 800 °C, and (**f**) 900 °C.

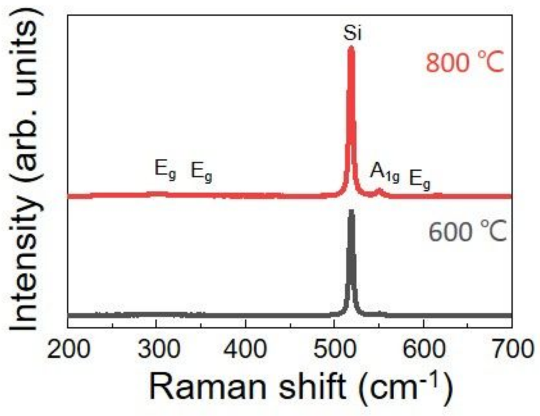

Figure S15. Raman spectra of Cr_2O_3 films synthesized at 600°C and 800°C.

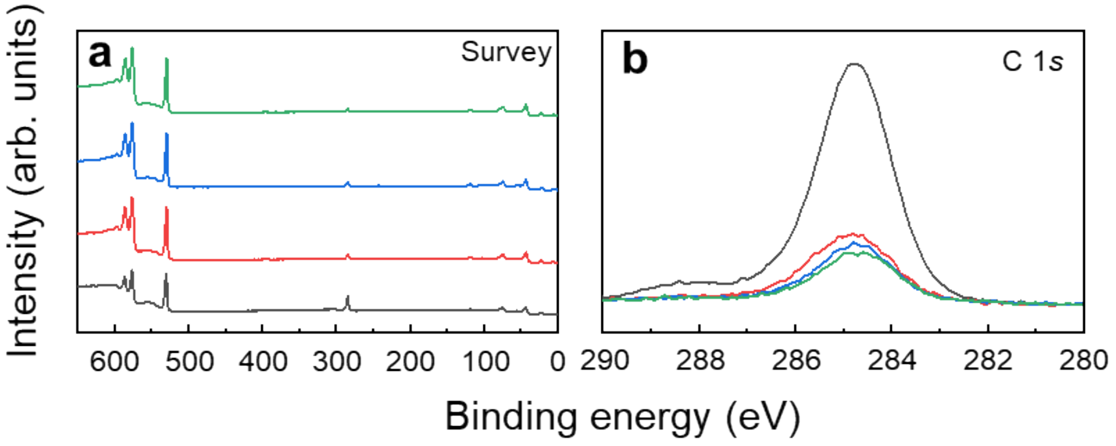

Figure S16. XPS depth profiling analysis of the Cr_2O_3 film synthesized at 900 °C: (**a**) survey spectrum, (**b**) C 1s depth profile.

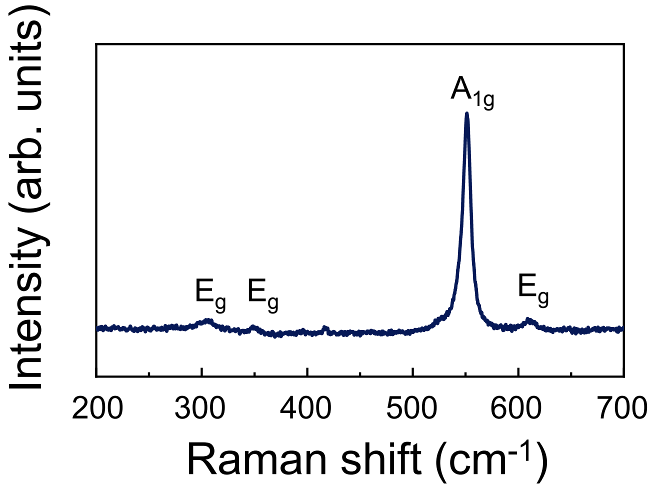

Figure S17. Raman spectra of Cr_2O_3 film on sapphire substrate

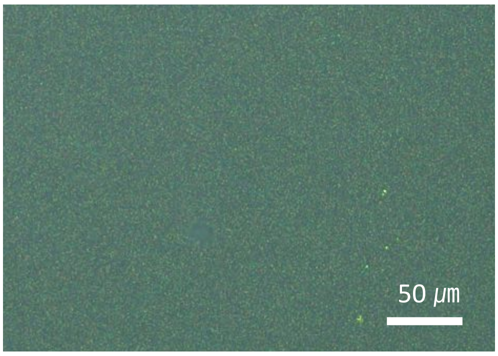

Figure S18. Optical image of Cr_2O_3 film on SiO_2/Si
